# A Comparison of the Colonic Microbiome and Volatile Organic Compound Metabolome of *Anoplocephala perfoliata* Infected and Non-Infected Horses: A Pilot Study

**DOI:** 10.3390/ani11030755

**Published:** 2021-03-09

**Authors:** Rachael Slater, Alessandra Frau, Jane Hodgkinson, Debra Archer, Chris Probert

**Affiliations:** 1 Institute of Systems, Molecular and Integrative Biology, University of Liverpool, Crown Street, Liverpool L69 3GE, UK; afrau@liverpool.ac.uk (A.F.); mdcsjp@liverpool.ac.uk (C.P.); 2 Institute of Infection, Veterinary and Ecological Science, University of Liverpool, Leahurst Campus, Chester High Road, Wirral CH64 7TE, UK; jhodgkin@liverpool.ac.uk (J.H.); darcher@liverpool.ac.uk (D.A.)

**Keywords:** anoplocephala perfoliata, equine, gut microbiome, volatile organic compounds (VOCs), omics integration

## Abstract

**Simple Summary:**

In horses, tapeworm infection is associated with specific forms of colic (abdominal pain) that can be life-threatening without surgical treatment. There is growing evidence that intestinal parasites interact with the gut bacteria, and the consequences of these interactions may influence the ability of the host to resist infection and parasite-associated disease. We aimed to compare the intestinal bacteria and the gases produced by metabolic processes in the gut between horses that had varying levels of tapeworms and those with no tapeworm present. Overall, the diversity of gut bacteria was similar in horses with and without tapeworms. There were some decreases in beneficial bacteria in horses with tapeworms, indicating a possible negative consequence of infection. Intestinal gases correlated with some bacteria indicating their functionality and use as potential markers of active bacteria. Our study validates further research investigating tapeworm and gut bacteria interactions in the horse.

**Abstract:**

*Anoplocephala perfoliata* is a common equine tapeworm associated with an increased risk of colic (abdominal pain) in horses. Identification of parasite and intestinal microbiota interactions have consequences for understanding the mechanisms behind parasite-associated colic and potential new methods for parasite control. *A. perfoliata* was diagnosed by counting of worms in the caecum post-mortem. Bacterial DNA was extracted from colonic contents and sequenced targeting of the 16S rRNA gene (V4 region). The volatile organic compound (VOC) metabolome of colonic contents was characterised using gas chromatography mass spectrometry. Bacterial diversity (alpha and beta) was similar between tapeworm infected and non-infected controls. Some compositional differences were apparent with down-regulation of operational taxonomic units (OTUs) belonging to the symbiotic families of Ruminococcaceae and Lachnospiraceae in the tapeworm-infected group. Overall tapeworm burden accounted for 7–8% of variation in the VOC profile (permutational multivariate analysis of variance). Integration of bacterial OTUs and VOCs demonstrated moderate to strong correlations indicating the potential of VOCs as markers for bacterial OTUs in equine colonic contents. This study has shown potential differences in the intestinal microbiome and metabolome of *A. perfoliata* infected and non-infected horses. This pilot study did not control for extrinsic factors including diet, disease history and stage of infection.

## 1. Introduction

The gastrointestinal tapeworm *Anoplocephala perfoliata* is a common inhabitant of the equine gut [1,2]. High levels of A. perfoliata infection are known to be risk factors for specific forms of colic (abdominal pain) in the horse including spasmodic colic, ileal impaction, caecal intussusception and caecal rupture [3,4,5,6,7] and some of these forms of colic may be fatal without surgical treatment. The exact mechanisms by which tapeworm-associated colic develops are not known; such knowledge would enhance our understanding of the pathophysiology of these and other forms of colic. Currently licenced anti-cestode drugs (pyrantel and praziquantel) appear effective at reducing burdens of *A. perfoliata*. However, anthelmintic resistance is a growing concern regarding the effective control of other equine parasites, such as strongyles and *Parascaris equorum* and may limit our ability to treat tapeworm infection in the future [8,9]. This is a particular concern given the small number of drug classes that are available to treat cestode burdens in horses [9,10]. This places greater emphasis on the need to better detect the presence of tapeworms and target treatments accordingly. Current diagnosis of tapeworm infection in live horses is based on serological or saliva antibody assays [11,12]. Studies have shown the antibodies may remain raised long-beyond clearance of infection, making them inaccurate for determining real-time infection [11,13,14]. Alternatively a faecal egg count (FEC) can be conducted, but this has only 61% sensitivity [15].

The horse is a hindgut fermenter and relies on volatile fatty acids (VFAs) produced by the microbiota to provide a large proportion of daily energy requirements [16,17]. Murine studies have shown evidence of the role of the commensal intestinal microbiota in the establishment and maintenance of parasites within the gut and this interaction has potential implications for host immunity [18,19,20]. Correlations between helminths and the faecal microbiota in live horses have been investigated using strongyle FEC as an indicator of infection [21,22,23]. In ponies predicted to be resistant or susceptible to strongyle infection, modest differences in microbiome composition were observed post-exposure to natural infection [21]. Another study demonstrated significant clustering in the microbial composition associated with FEC rather than anthelmintic treatment, but there were no differences in faecal bacterial diversity between horses with high and low FECs [22]. Both studies reported an increase in members of the phylum Proteobacteria in horses with high FECs. Three studies in horses have shown a shift in microbial composition following anthelmintic treatment, irrespective of whether FECs were high or low [23,24,25]. Differences in microbial composition following treatment may also be more evident in young animals [23,24]. These results indicate a possible effect of anthelmintic medication, or clearance of strongyles (even at low levels) from the gastrointestinal tract, on the faecal microbiome in some populations of horses. Collectively, these initial studies imply that intestinal helminth-microbiota interactions occur in the horse and warrant further study in other equine species of parasite, including cestodes. The relationship of *A. perfoliata* with the gut microbiome and metabolome (functional microbiome) of the horse has not previously been investigated. Volatile organic compounds (referred to as the VOC metabolome) may be generated by physiological processes from the host or the microbiota [26,27]. In the horse, VOC patterns appear to mirror the faecal microbiota [28]. Studies incorporating both microbiome and metabolome interactions with parasites are important in understanding the functional effect parasites have on the gut microbiota and intercommunications within the host [20]. The knowledge of such interactions may have beneficial consequences for equine health through alternatives to parasite control [29].

We hypothesised that the colonic microbiome and VOC metabolome would differ between *A. perfoliata*-positive and -negative horses. To enable accurate classification of tapeworm-positive and -negative horses, we collected samples post-mortem from abattoir material (gold-standard diagnosis of *A. perfoliata*). Collection of abattoir material enabled colonic contents to be taken for microbiome and metabolome analysis rather than using a faecal proxy. Faeces has been shown to be representative of the distal regions of the hindgut but is less representative of more proximal regions [30,31]. The aims of this study were to compare the microbiome and VOC metabolome of the distal colonic contents of *A. perfoliata* infected and non-infected horses and those with high and low strongyle FECs, to rule this out as a confounding factor. We also aimed to investigate correlations between 16S rRNA sequence data and VOCs to infer active species and functionality of the microbiome that may be associated with tapeworm infection.

## 2. Materials and Methods

### 2.1. Sample Collection

Samples were collected from 51 horses slaughtered for non-experimental purposes at an abattoir in the South of the UK in November 2015. A power calculation for this pilot study was not performed as, to the authors’ knowledge, this was the first study to compare the gut microbiome of tapeworm infected and non-infected horses. However, studies investigating strongyle species and microbiome interactions sampled similar numbers of horses to those used here [21,22,24]. Metadata including previous diet, breed, anthelmintic treatment and medications history were not available to be recorded. Specific horse age was also not available, but no pre-weaned foals were included. Post-mortem (immediately after slaughter and removal of the viscera—within 15 min) the caecum was opened to expose the caecal mucosa and visual assessment was used to estimate numbers of *A. perfoliata* present (gold standard). Samples were categorised as low (1–20 tapeworms, n = 7), medium (21–49, n = 5), high (≥50, n = 5), tapeworm positive but numbers unrecorded (n = 4) and tapeworm negative (n = 30). The low threshold was chosen based on evidence that small numbers of tapeworms (1–20 worms) cause little intestinal pathology [32]. The thresholds set for the medium and high categories were arbitrary; they were chosen to demonstrate patterns with increased worm burden as there is a positive correlation between infection intensity and the risk of tapeworm associated colic [4]. For microbiome and metabolome analysis luminal contents (~25 mL) were collected from the large colon (pelvic flexure) for consistency in sampling. The regions of the hindgut where the tapeworms were located (caecum) were empty in many horses, possibly because of withholding of feed prior to slaughter. Colonic contents were transferred on dry ice to the laboratory and stored at −80 °C. Samples of rectal contents (~10 g) were also collected into universal containers and transported in a cool box and stored at 4 °C upon reaching the laboratory. Strongyle and ascarid worm faecal egg counts (FEC) (recorded in Table S1) were performed on rectal contents using a centrifugal floatation technique with a detection limit of one egg per gram (e.p.g.) within 10 days of collection [33]. Frozen colonic contents (~5 g) were freeze-dried for 48 h (Edwards High Vacuum, West Sussex, UK). Two aliquots of freeze-dried colonic-contents were created, one for VOC profiling (100 mg in a glass headspace vial) and one for DNA extraction (100 mg). For DNA extraction two glass beads (4 mm, undrilled, Fisher Scientific, Loughborough, UK) were added before the vials were placed in a frozen rack (−80 °C) and bead-beaten (TissueLyser II, Qiagen, Manchester, UK) for 2 min at full power (30 Hz). Freeze-drying of faecal and colonic contents prior to bead-beating has previously been applied in equine studies to disrupt the fibrous material [31,34].

### 2.2. Microbiome Profiling: 16S rRNA Sequencing

DNA extraction was performed on freeze-dried samples using Qiagen QIamp DNA stool mini kits (Qiagen, Manchester, UK) and steps were followed according to the manufacturer’s instructions. After the addition of ASL buffer (Qiagen, Manchester, UK) to the sample and briefly vortexing, it was incubated in a heat block at 95 °C for 5 min, as suggested by the manufacturer. To amplify the bacterial 16S rRNA gene (V4 region), a universal tail tag dual indexing barcode approach was used [35]. For the first round of PCR the primers used were F515 (NNN NNG TGC CAG CMG CCG CGG TAA) and R806 (GG ACT ACH VGG GTW TCT AAT) (HPLC grade, Integrated DNA Technologies, Coralville, IA, USA) [36]. A PCR master mix was made containing (per total 20 µL reaction): 1× Q5 reaction buffer, 0.02 U/µL Q5 High-Fidelity DNA polymerase, 200 µM dNTPs (NEB), 0.125 µM of each forward and reverse primers made up to 15 µL with molecular-grade water. The master mix was divided between wells, with 5 µL (1 ng/µL) of template DNA. The plate was loaded into a pre-heated thermocycler (Multigene^™^ and Multigene^™^ OptiMax, Labnet International, Edison, NJ, USA) with an initial denaturation of 2 min at 98 °C, followed by 14 cycles of 20 s at 95 °C, 15 s at 65 °C, 30 s at 72 °C. There was a final extension step of 5 min at 72 °C and then samples were held at 4 °C until purification (within 24 h). The first round of PCR was carried out in duplicate to reduce PCR bias. Purification was performed using the AxyPrep Mag PCR Clean-up kit (Axygen, Corning, NY, USA) and the entire eluate (10 µL) was used as template for index PCR. Index PCR was carried out in a volume of 20 µL with the same reagents and method used in the first round of PCR. An exception was the use of a set of barcoded index primers (listed in Table S2, TruGrade, Integrated DNA Technologies, Coralville, IA, USA) used at 0.25 µM per sample and 15 PCR cycles. Index PCR was carried out in duplicate, and purification was repeated on the pooled duplicates. The final elute was 25 µL and the concentration of DNA was recorded using a Qubit (Qubit dsDNA HS assay kit, Life Technologies, Carlsbad, CA, USA). Sample pooling was performed based on sample concentration and fragment size determined by a bioanalyzer (2100 Bioanalyzer, Agilent Technologies, Santa Clara, CA, USA) by the Centre for Genomic Research (CGR), Liverpool, UK. Paired-end (2 × 250 bp) sequencing was performed on an Illumina MiSeq platform by the CGR, Liverpool, UK.

### 2.3. Volatile Organic Compound (VOC) Metabolome Profiling

Aliquots of freeze-dried colonic contents were analysed by headspace solid-phase microextraction gas chromatography mass spectrometry (HS-SPME-GCMS). Freeze-dried colon contents were taken forward for analysis rather than wet samples because greater numbers of VOCs were detected (results in Supplementary: Figure S1). The SPME fibre coating used was divinylbenzene-carboxen-polydimethylsiloxane (DVB-CAR-PDMS) 50/30µm (1 cm). Samples were heated at 60 °C for 30 min prior to SPME fibre exposure to the sample headspace for 20 min. GCMS conditions were as described previously [37].

### 2.4. Data Analysis

#### 2.4.1. Microbiome

**Figure 1:**
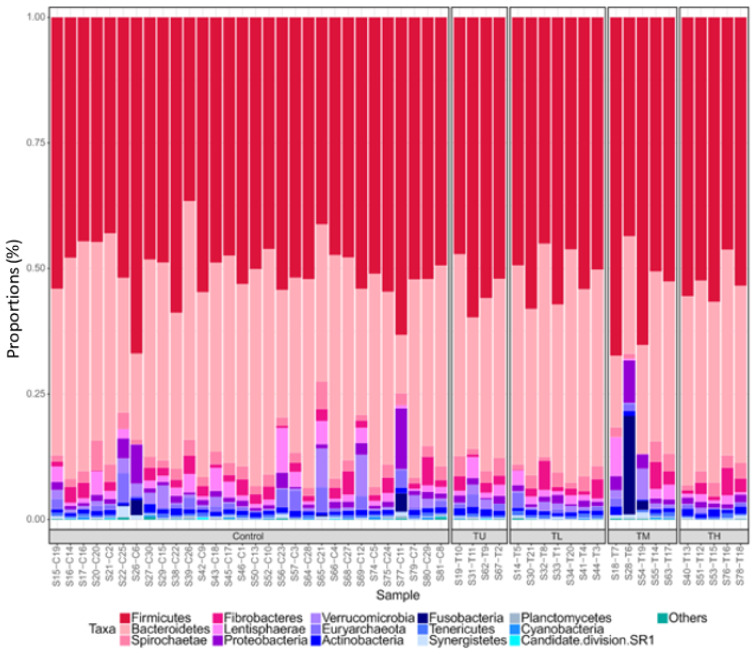
Taxonomic summary at phylum level for all samples. Key: TU = tapeworm positive with burden unrecorded, TL = low tapeworm (1–20 worms), TM = medium tapeworm (21–49 worms), TH = high tapeworm (≥50 worms).

Illumina adapter sequences were trimmed from raw FastQ files using Cutadapt (1.2.1) [38]. The reads were further trimmed using Sickle (1.2) [39] with a minimum window quality score of 20. Reads shorter than 200 bp after trimming were removed. Samples with fewer than 1000 reads were also removed at this stage. Sequence clustering (99% similarity) was performed by SWARM 2.0 (d = 3) [40] and chimeras were filtered out using UCHIME [41]. The following steps were then carried out using Quantitative Insights Into Microbial Ecology (QIIME) (1.9.1) [42]. The classifier tool BLAST [43] was applied together with the SILVA (SILVA_123) database [44] to assign OTUs at a 99% threshold. The final steps were alignment of OTUs with the database using PyNAST [45] and construction of a phylogenetic tree using FastTree [46]. Prior to statistical analysis three samples were excluded. One sample (C25) failed to freeze-dry and two samples (T6 and C11) contained higher relative abundances of Fusobacteria and Proteobacteria than Bacteroidetes (the second most dominant phyla in all other samples) (Figure 1). The demographics of the animals in this study were unknown, and therefore these samples were removed to prevent bias. Removal of outliers using these criteria has been applied in equine faecal microbiome studies previously [22]. In R (version 3.5.1) the following statistical analysis was performed using methods described previously [47]. Specifically, a taxonomy plot was constructed at phylum level. The data were normalised to relative abundance for these visualisation plots. Numbers of horses with low (n = 7), medium (n = 4) and high (n = 5) tapeworm burdens were considered too small to compare to controls (CO, n = 28) by statistical analysis. Therefore, groups to be compared by statistical analysis were divided as follows: all tapeworm positive horses (TP, n = 20) were compared to CO and horses with clinically important burdens (≥21 tapeworms) referred to as TP_21 (n = 9) were also compared to CO. Diversity indices were calculated at OTU level using the R package vegan [48]. The alpha diversity (Richness, Shannon and Fisher indices) was calculated for each group and differences between groups were compared using the aov() function in R. The beta diversity was plotted on non-metric multidimensional scaling (NMDS) ordination plots (distances: Bray-Curtis, Unifrac and Weighted Unifrac). Statistical differences in the beta diversity between groups was assessed by permutational multivariate analysis of variance (PERMANOVA) using the adonis() function. Differential abundance of taxa (phylum, order, class, family, genus and OTU level) between CO and tapeworm groups (TP and TP_21) was evaluated using the DESeq2 R package [49]. To investigate the association of strongyle FEC in this cohort of horses alpha and beta diversity indices and the DESeq R package were applied to assess differences in the microbiota of groups of horses with low ≤10 e.p.g (n = 10, Low_FEC) or high ≥100 e.p.g (n = 24, High_FEC) strongyle FEC, as investigated previously [22]. A PERMANOVA (gower distance) model to access the variation described by tapeworm burden and FEC in the gut microbiome was performed using the vegan R package, function adonis2().

#### 2.4.2. Volatile Organic Compound (VOC) Metabolome

VOC data were processed using Automated Mass Spectral Deconvolution System (AMDIS-version 2.71, 2012, www.amdis.net, (accessed on 29 November 2020)) coupled to the National Institute of Standards and Technology (NIST) mass spectral library (version 2.0, 2011, purchased from PerkinElmer, Beaconsfield, UK) to putatively identify VOCs. The R package Metab [50] was used to align data. Low quality data points were removed using the criteria that a VOC must be present in at least 50% of samples within at least one condition to remain present. Following this, half-minimum values were imputed to replace missing data as performed previously [28]. Statistical analysis of VOC metabolome data was performed on the same groups as the microbiome data. The number of VOCs and differences in their abundance between tapeworm groups (TP and TP_21) and CO was evaluated by an independent *t*-test. To evaluate VOC abundance associated with high and low FECs an independent *t*-test was performed on the same groups outlined in the microbiome analysis. The same PERMANOVA model in the microbiome analysis including FEC and tapeworm burden was applied to the VOC metabolome data.

### 2.5. Integration of Omics Data

The R package mixOmics using the Data Integration Analysis for Biomarker discovery (DIABLO) framework was used to identify correlations between OTUs and VOCs. Specifically, the N-integrative supervised analysis was performed with DIABLO [51]. Prior to input of data into the DIABLO model, 16S rRNA data were normalised by Total Sum Scaling normalisation (TSS) followed by centred log-ratio (CLR). VOC data were normalised as described in Section 2.4. The first step involved fitting the model prior to variable selection to assess performance (based on correct assignment of groups) using the function perf(). A 10-fold cross validation of perf() was undertaken. From the cross-validation, a performance plot was constructed to calculate the balanced error rate (BER). The balanced error rate calculates the average proportion of wrongly classified samples in each class, weighted by the number of samples in each class. Based in the performance of the model the optimal number of components was chosen using the function choice.ncomp(). Next, the tune.block.splsda function was used to choose the optimum number of variables for each type of omics (OTUs and VOCs). Using these optimal variables, the final DIABLO model was constructed. A scatterplot (Pearson’s correlation) was generated for OTUs and VOCs separately to evaluate which omics was the most important in distinguishing between time points using the function plotIndiv(). The cor.test() function (for paired data) was used to determine significant pair-wise correlations identified between OTUs and VOCs. P values were adjusted by FDR. A plot generated by the plotDIABLO() function was used to show overall correlation between selected OTUs and VOCs. For visualisation of correlations between specific OTUs and VOCs a heatmap was constructed using the plotVar() function. Integrated analysis was not performed on the TP_21 sub-set or FEC groupings because of the small sample sizes.

## 3. Results

### 3.1. Microbiome Profiling

The total number of sequences was 6,186,806 after filtering. The sequences clustered to a total number of 1,458. There was a minimum of 75,244 sequences per sample and a maximum of 167,089; there were 116,212 sequences per sample on average. In total, 19 phyla were identified, and the most abundant phyla were Firmicutes (51.6%), Bacteroidetes (36.1%), Spirochaetae (2.3%) and Fibrobacteres (2.0%) (Figure 1).

**Figure 2:**
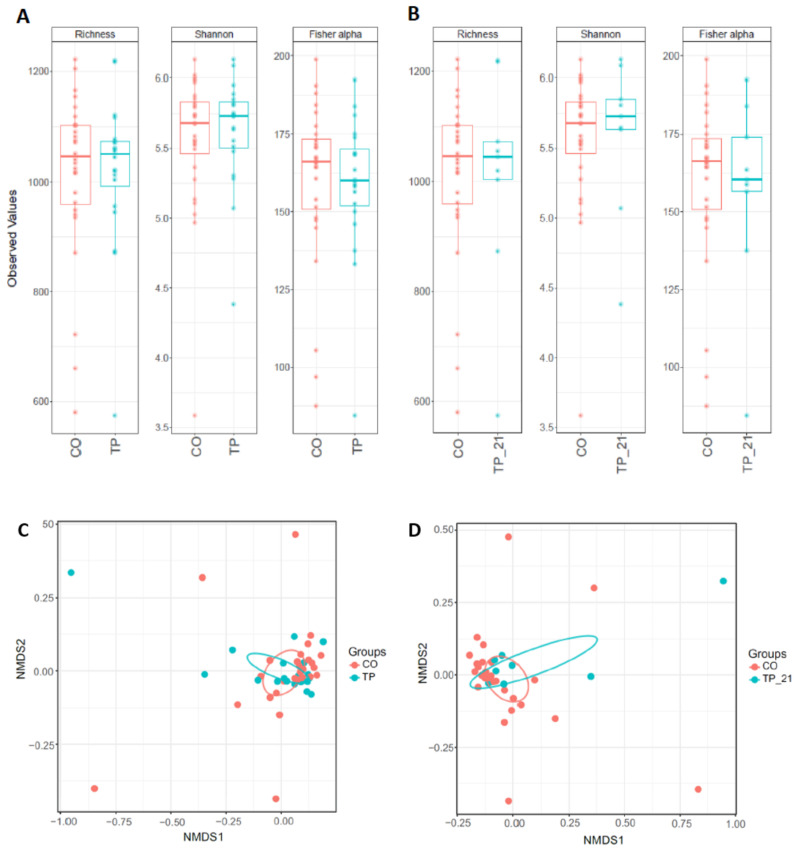
Alpha (**A**,**B**) and beta diversity (Bray-Curtis distance is shown) (**C**,**D**) indices (OTU level) of the colonic contents of horses infected with *Anoplocephala perfoliata* and non-infected controls. Alpha diversity was not significantly different between groups, pair-wise ANOVA (*p* > 0.05). The beta diversity between groups was not significant (PERMANOVA, *p* > 0.05). In (**A**,**C**) CO (n = 28) vs. TP (n = 20), (**B**,**D**) CO vs. TP_21 (n = 9). Key: TP = tapeworm positive (n = 20), TP_21 = tapeworm samples with >21 worms (n = 9), CO = control (tapeworm negative), NMDS = non-metric multidimensional scaling.

**Figure 3:**
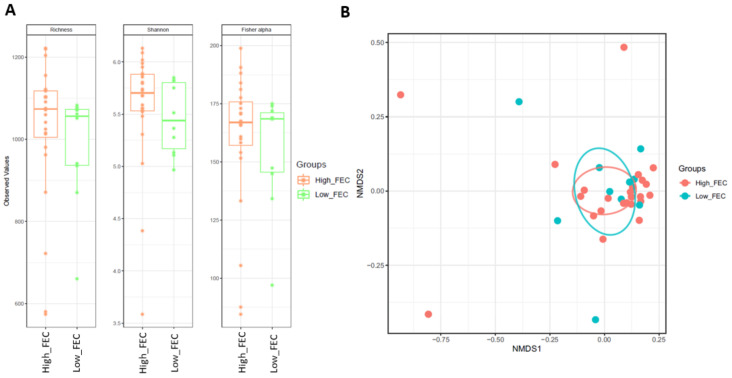
Alpha (**A**) and beta (**B**) (Bray-Curtis distance is shown) diversity indices for horses with low (<10 e.p.g) and high (>100 e.p.g) strongyle FECs. Alpha diversity was not significantly different between groups, pair-wise ANOVA (*p* > 0.05). The beta diversity between groups was not significant (permutational multivariate analysis of variance, *p* > 0.05) Key: High_FEC = high strongyles (>100 e.p.g) n = 24, Low_FEC = low strongyles (<10 e.p.g) n = 10, FEC = faecal egg count, e.p.g = eggs per gram, NMDS = non-metric multidimensional scaling.

Box plots of the alpha diversity indices (Richness, Shannon and Fisher alpha) between tapeworm and control groups are shown in Figure 2A,B. Pair-wise comparisons between groups were not significant (*p* > 0.05). NMDS plots of the beta diversity (Bray-Curtis) of tapeworm and control groups are shown in Figure 2C,D and demonstrated no distinct clustering. PERMANOVA of beta diversity values (Bray-Curtis, Weighted Unifrac, Unifrac) revealed there were no significant differences in beta diversity between groups for both TP vs. CO and TP_21 vs. CO. Box plots of the alpha diversity indices (Richness, Shannon and Fisher alpha) and NMDS plots for beta diversity (Bray-Curtis) for comparisons between Low_FEC and High_FEC are shown in Figure 3. There were no significant differences in alpha (pair-wise ANOVA, *p* > 0.05) or beta (PERMANOVA, *p* > 0.05) diversity between Low_FEC and High_FEC groups.

Using the DESeq2 R package, there were no significant differences in taxa abundance at phylum, order or class levels between tapeworm and control samples. Some differences at family and genus level were observed and are listed in Table 1. At OTU level, 69 OTUs (Figure S2A) were found to be significantly different between TP and CO; of these, 17 (24.6%) were more abundant in the TP group. When TP_21 and CO were compared, significant differences in taxa between groups were observed at order, family, genus (Table 1) and OTU level. One hundred and eighteen OTUs (Figure S2B) were significantly different with 28 (23.7%) more abundant in the TP_21 group. Taxa differential analysis revealed significant differences at order, family, genus (Table 1) and OTU level between Low_FEC and High_FEC samples. Ninety-eight OTUs were significantly different with 76 (77.6%) more abundant in the High_FEC group (Figure S3). A PERMANOVA model, which included TP and CO samples, revealed tapeworm burden accounted for 3% (p = 0.03) of variation and strongyle FEC accounted for the same, 3% (*p* = 0.04). When the PERMANOVA model included TP_21 and CO samples, 4% (*p* = 0.04) of variation in the VOC profile could be described by tapeworm burden but no significant variation could be described by strongyle FEC (4%, *p* = 0.10).

### 3.2. Volatile Organic Compound (VOC) Profiling

Across all samples, 85 VOCs were identified. A complete list with the VOCs identified within TP and CO samples is shown in Table S3. A significantly higher mean number of VOCs was detected in the TP group (mean 72 SE ± 1.44) compared to CO (67 ± 1.21) (*p* = 0.03, *t*-test). For the TP_21 group the mean number of VOCs (67 ± 2.43) was not significantly different from CO (72 ± 1.21, *p* = 0.12, *t*-test). Principal component analysis did not reveal any distinct clustering of groups Figure S4A,B.

**Figure 4:**
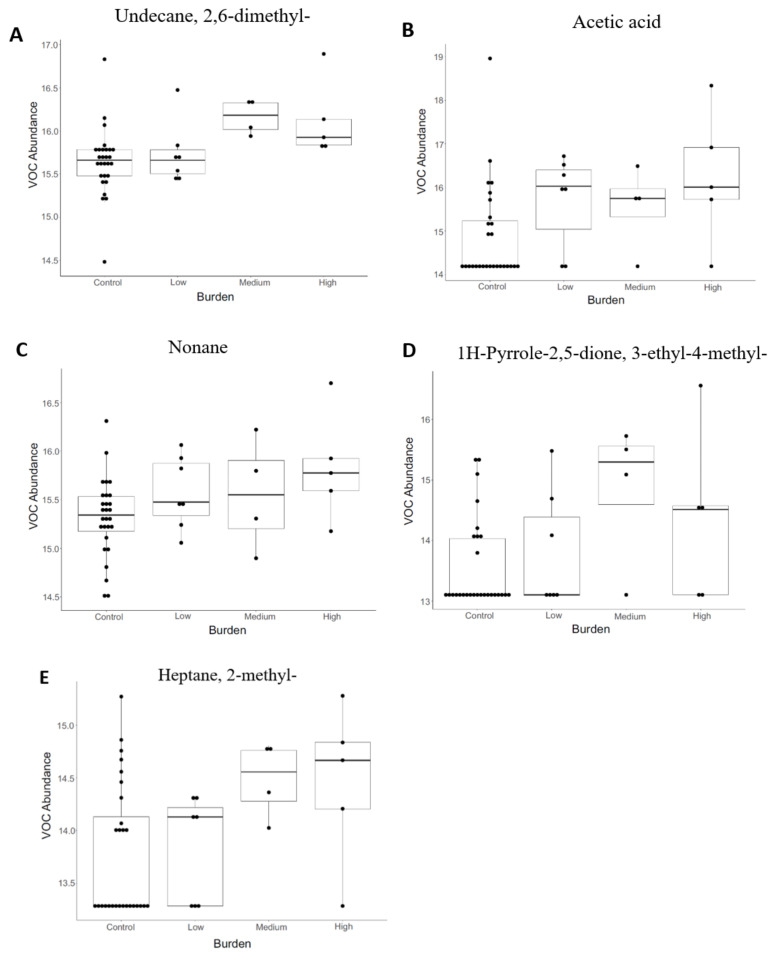
Box and whisker plots of VOC abundance of the colonic contents of horses infected with *Anoplocephala perfoliata* and non-infected controls. The three volatile organic compounds (VOCs) with the smallest *p*-values, identified by *t*-test for both TP vs. CO (**A**–**C**) and TP_21 vs. CO (**A**,**D**,**E**) are shown. Plots were constructed to show VOC abundance change in individual groups: control, low (1–20 worms), medium (21–49 worms) and high (≥50 worms) to show gradient change of compounds with level of tapeworm burden.

In total, 24 VOCs were significantly different in abundance between TP and CO (*p* < 0.05, *t*-test, pre-correction for multiple comparisons) (Table 2). For TP_21 and CO, 17 VOCs were significantly different in abundance between groups (*p* < 0.05, *t*-test, pre-correction for multiple comparisons) (Table 2). For visualisation purposes, the 3 VOCs with the smallest *p*-values from both tapeworm groups were plotted as box and whisker plots in Figure 4. For strongyle FEC, there were two VOCs in significantly greater abundance in the High _FEC group (*p* < 0.05, *t*-test, pre-correction for multiple comparisons) (Table 2). However, after correction for multiple comparisons, none of the VOCs were significantly different in abundance between horses with tapeworm and controls or between those with high and low strongyle FECs. When TP and CO were included in a PERMANOVA model, tapeworm burden explained 7% (*p* = 0.01) of variation and strongyle FEC was not able to explain any variation (2%, *p* = 0.23). The inclusion of TP_21 and CO in a PERMANOVA model resulted in 8% (*p* = 0.02) of variation in the VOC profile explained by tapeworm burden and no effect of strongyle FEC (2%, *p* = 0.55).

### 3.3. Integrated-Omics

**Figure 5:**
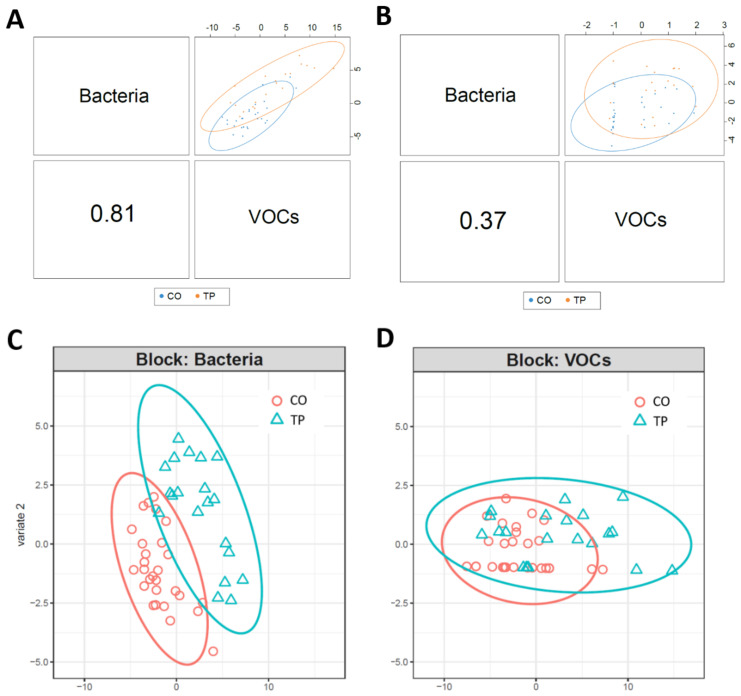
A Pearson’s correlation plot of bacteria and volatile organic compound (VOC) data of the colonic contents of horses infected with *Anoplocephala perfoliata* and non-infected controls. Component 1 is shown in (**A**) and component 2 is shown in (**B**). The ability of the model to separate CO and TP by bacteria alone is demonstrated by plot (**C**), and VOCs alone by plot (**D**). Key: TP = tapeworm positive (n = 20), CO = control (tapeworm negative, n = 28).

**Figure 6:**
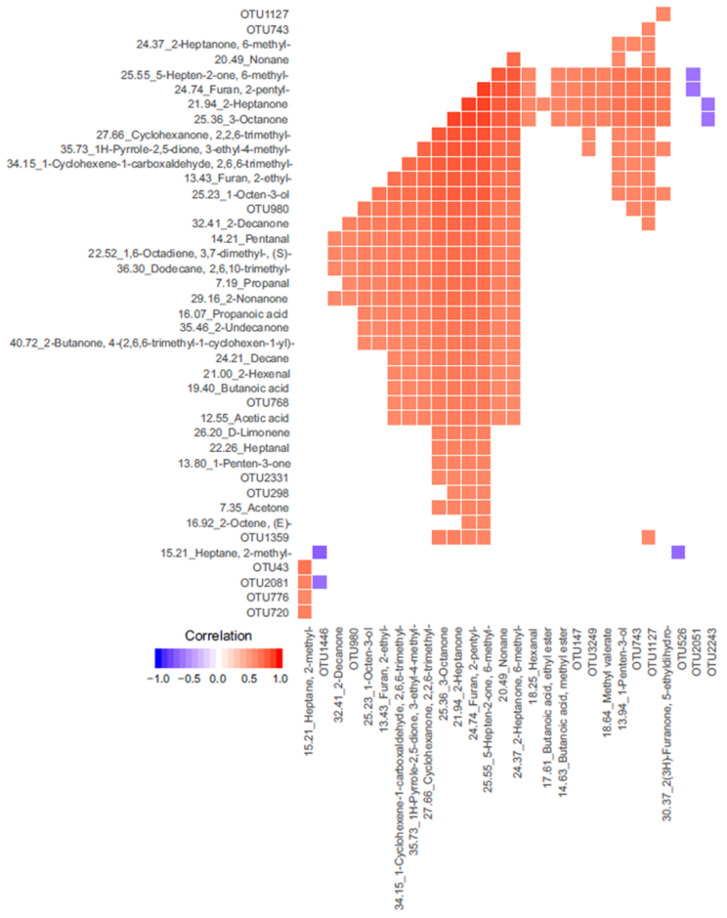
A heatmap of correlations (sorted by correlation values) between bacterial operational taxonomic units (OTUs) and volatile organic compounds (VOCs) found in the colonic contents of horses infected with *Anoplocephala perfoliata* and non-infected controls. Details of OTU taxonomic identification are in Table S4.

The DIABLO model generated a balanced error rate of 35–44% (Figure S5). A Pearson’s correlation plot demonstrated a strong correlation between bacterial OTUs and VOCs, but the combination of these omics resulted in only subtle clustering of TP and CO (Figure 5A,B). Overall, bacterial OTUs were better at separating TP and CO groups than VOCs (Figure 5C,D). A total of 1257 correlations (0.3 and above) were identified between OTUs and VOCs. Correlations identified using the plotVar() function are shown in a heatmap in Figure 6. To assess the use of VOCs as markers for OTUs in higher or lower abundance in control or tapeworm samples identified in single omics, a table of VOCs and OTUs which were significant in the single omics analysis (pre-FDR corrected) and had significant correlations in the integrated analysis was constructed (Table 3). Of interest, OTU2331 and OTU147 (both Prevotellaceae) were in higher abundance in TP in single omics analysis and each positively correlated (0.36–0.6) with 17 and 19 VOCs respectively, which were also in significantly greater abundance in TP in single omics analysis. OTU2051 (Rikenellaceae), identified in higher abundance in the CO group, was found to be negatively correlated (−0.6) with furan,-2-pentyl and 5-hepten-2-one-6-methyl.

## 4. Discussion

This is the first study to investigate the intestinal microbiome and metabolome of horses with and without infection of the important equine parasite *A. perfoliata*, based on the gold standard of physical identification of parasites at post-mortem. Knowledge about whether tapeworm infection is associated with changes in the microbiome and metabolome of the horse may further our understanding of the parasite-microbiota interactions in the equine gut and help us to better understand the pathophysiology of tapeworm-associated colic.

The present study found that Firmicutes (51.6%) and Bacteroidetes (36.1%) were the dominant phyla in the samples obtained, which is consistent with other equine studies [21,52,53]. The bacterial diversity in samples from horses that were either tapeworm positive or negative (CO group) were similar, a finding consistent with many other studies comparing the gut microbiome of parasite infected and non-infected animals [21,22,54,55,56,57]. Significant differences in microbiota diversity in parasite positive and negative animals have been more widely reported in rodent and rabbit studies in which the level of infection and diet can be closely controlled and larger sample sizes can be used [58,59,60,61], and which is something that is difficult to practically achieve in studies of horses, which are usually client-owned rather than research horses.

Here, a decreased abundance of some fibrolytic bacteria considered symbiotic, including an unidentified genus belonging to the family Ruminococcaceae UCG-004, was observed in the tapeworm groups when compared to the controls [62,63]. Fewer Ruminococcaceae were also seen in rabbits and pigs (genus level, *Ruminococcus*) infected with intestinal parasites [60,64]. A decrease in *Ruminococcus* was previously identified as a consequence of strongyle infection in ponies identified as being susceptible [21]. The authors of the latter study suggested that a reduction in butanoic acid (because of a reduction in *Ruminococcus*) could influence the level of inflammation caused by infection. However, the authors did not measure butanoic acid, and in the present study, a correlation between butanoic acid and symbiotic bacteria was not observed. The role of symbiotic bacteria during parasite infection requires further investigation to determine if interactions occur as a result of, or as a pre-cursor to infection, and what consequences this may have for the host.

The genus *Selenomonas 3* was more abundant in the tapeworm positive (TP_21) group compared to non-tapeworm infected controls (CO). Little is known about *Selenomonas.* Previous studies have found that *Selenomonas ruminatum* was more abundant in goats infected with the gastrointestinal parasite *Haemonchus contortus* [55]. Some *Selenomonas* species have also been associated with inflammation in human patients with periodontal disease [65]. Species of *Selenomonas* are involved in the fermentation pathway of starch and sugars in the hindgut so an increased presence may be attributable to the diet or to an adaption of the host or gut microbiota to compete for nutrients with the parasite [66]. In the current study, just two out of nine horses in the TP_21 group had high levels of *Selenomonas 3*.

In general, as tapeworm burdens increase, severity of lesions, inflammation of the mucosa and the risk of colic increases [4,32]. However, fewer tapeworms are required to produce severe lesions at the ileo-caecal junction than the caecal wall [67]. Therefore, numbers of tapeworms may not have been fully representative of mucosal inflammation in this work, and the specific site and extent of inflammation was not recorded. Future studies should record sites of parasite attachment as well as taking mucosal samples from inflamed and non-inflamed regions, furthermore mucosal samples from controls would also help to determine parasite related and non-parasite related inflammation. A decrease in bacterial diversity, as well as an increase in inflammatory biomarkers, has been reported in horses post-treatment for cyathostomins [24]. Post-dosing colic has been linked to high ELISA optical densities for tapeworm, suggesting colic may be a result of the removal of significant tapeworm burdens [68]. The role of the gut microbiota and tapeworm in the development of or reduction in likelihood of tapeworm-associated equine colic remains unclear, but the subtle changes in the gut microbiota observed in this work warrant further study in this area. Sampling from gut regions where tapeworm attachment and associated gastrointestinal pathology (e.g., ileo-caecal junction and caecal wall) may provide further insight into whether parasite-microbiota interactions have a role in specific types of equine colic.

A PERMANOVA analysis revealed that tapeworm burden explained a small amount (7–8%) of variation in the VOC profile, but evidently, this was not enough to demonstrate clear clustering for tapeworm in PCA analysis. The VOC metabolome changed little with the FEC, and only two VOCs were significantly different in samples with high FEC (prior to FDR). There was no significant variation described in the PERMANOVA analysis. These findings were similar to a previous study, using a different metabolomics platform, where only moderate differences in faecal metabolite profiles were observed between horses with high and low FECs [23]. Other factors, which have been described previously to influence the equine hindgut microbiome and metabolome, including horse age, breed, diet, and history of gastrointestinal disease, as well as antibiotic and anthelmintic treatment [25,62,69,70,71], were not recorded in the current study. These factors may have been responsible for other variations observed in the VOC profile. The inability to record this metadata was the main limitation of this post-mortem study. For future investigations, to maximise the ability to determine variation influenced by parasites alone on the microbiota, extrinsic factors should be controlled for. However, this information may be difficult to obtain for horses submitted to an abattoir which, at present is the most practical method for sampling large numbers of horses using the gold standard for equine tapeworm diagnosis (direct evaluation of parasite status in the gastrointestinal tract post-mortem).

Integrated analysis demonstrated a strong correlation (0.81) between bacterial OTUs and VOCs. In the single omics analysis 69 OTUs were found to be significantly different between TP and CO. Of these, 10 were correlated with VOCs. A lack of correlation for the other 59 OTUs with VOCs could imply the organisms were not active, as DNA-based studies are not representative of active species [72]. Furthermore, the use of one metabolomics platform and extraction technique will not encompass all metabolites within a biological matrix so there is a possibility that metabolites directly correlating with the most abundant organisms were not detected here [73].

There were no significantly differences in abundance of VOCs between tapeworm infected and non-infected controls after *p*-values were adjusted for multiple comparisons. Despite not reaching statistical significance in the single omics analysis many of these VOCs were responsible for the variation shown in the Pearson’s correlation plot (supervised analysis) and were correlated with several OTUs. This demonstrates that, although statistical significance was not reached after correction for multiple comparisons, these VOCs were still important in discriminating between tapeworm infected and non-infected controls when combined with other analysis and the study may have simply been under-powered when evaluated as a single omics. Furthermore, the use of supervised analysis in noisy datasets with confounding factors such as this, may be advantageous [74]. However, when supervised analysis is employed, it is important to check that the data are not being over fitted. In the present study the BER was used to evaluate the supervised model for overfitting. We achieved a BER of 35–44%, an indication that the group separations observed between TP and CO should be interpreted with caution [75]. The high error rate may be because of the small sample size (more features than samples were included in the model) or because the differences between the two groups were not enough to accurately classify the samples.

Several VOCs in higher abundance in TP samples were correlated with two OTUs (OTU2331 and OTU147) belonging to the family Prevotellaceae. Members of the Prevotellaceae family have been associated with driving chronic intestinal inflammation in mice with inflammasome-mediated dysbiosis [76,77]. It may be reasonable to speculate that the more highly abundant OTUs were associated with parasite-driven inflammation. On the other hand, some of the correlating VOCs, including 2-heptanone, 1-octen-3-ol, 2-octanone and furan compounds, have previously been identified as being of possible fungal origin [78,79]. Furthermore, in healthy human subjects consuming a high-carbohydrate diet, a correlation was observed between the fungal genus *Candida* and members of Prevotellaceae (genus *Prevotella)* [80]. The authors speculated that *Candida* were able to break down complex carbohydrates into simple sugars to be fermented by *Prevotella*, to produce acetic acid. In the current work acetic acid was also more abundant in the tapeworm group, supporting this hypothesis. Fungal populations were not characterised in this study, so it can only be speculated that these VOCs were related to fungal metabolism. In addition, the OTUs of interest could not specifically be identified as *Prevotella*, but as members of the family Prevotellaceae, in which there are three other possible genera to which the OTUs may have belonged. In horses, this is one of the few studies to correlate findings of a 16S rRNA study with the gut metabolome in a statistical model. The integration of omics to address the same hypothesis was able to provide stronger evidence for the conclusions made. A larger sample size is required to clarify whether the combination of bacterial OTUs and VOCs can accurately distinguish between horses with and without tapeworm infection.

A comparison between horses with high and low strongyle FECs was performed to evaluate the association of FEC with the gut microbiome and metabolome as a potential confounding factor in this study. Here, we categorised horses into low and high FEC groups, as performed previously by others [22,23]. There was no significant association between the strongyle FEC and the overall gut microbiota diversity, as observed previously in faeces [21,22]. Differential analysis demonstrated some significant differences in microbial taxa at order, family and genus level between horses with high and low FECs that were not shared with tapeworm and control comparisons. An increase in members of the phylum Proteobacteria was observed in horses with high or a susceptibility to a high strongyle FEC [21,22], but this was not observed in tapeworm infected horses or in those in the High_FEC group in the present study, despite a larger sample size. However, the order Rhodospirillales, family and genus *Thalassospira* (all members of phylum Proteobacteria) were more abundant in those with High_FEC compared to Low_FEC. Possibly the levels of infection were not high enough to enable differences to be observed at phylum level between groups or other confounding factors of the current study, including mixed infections, a lack of control for diet, age, breed and anthelmintic and antibiotic treatment history. To date, differences in the microbiota between horses with high levels of mixed parasite burdens with those with low mixed burdens has not been compared and was not possible in the current study because of small sample sizes. However, investigating the impact of general high parasite burdens on the equine microbiota and overall health would be interesting and may our further knowledge of parasite-microbiota interactions in the horse. It should be noted that FEC and numbers of parasites present in the gastrointestinal tract have a weak correlation [81]. A validated serum ELISA for encysted cyathostomins has become available since the samples were collected for this study [82,83]. Future studies should aim to use this alongside other diagnostic methods.

Understanding the mechanisms responsible for microbiota, parasite and host interactions were beyond the scope of this study. Whether *A. perfoliata* infection and the gut microbiota interact to produce positive (stimulation of the immune system) or negative (a role in the development of colic) impacts on the host remains unknown. Others have proposed the idea that the use of probiotics and prebiotics to manipulate the gut microbiota may help control gastrointestinal parasites as an alternative to drugs [84]. However, before further exploration is performed, the wider impacts of such therapies on the host should be considered.

## 5. Conclusions

This is the first study to integrate microbiome and metabolome data in relation with the tapeworm status of the horse. Furthermore, the microbiome and metabolome of gut contents have not previously been reported in previous equine parasitology studies. We found statistically significant correlations between specific OTUs and the presence of tapeworm. Some VOCs and bacterial OTUs were also correlated. The relationship between VOCs and tapeworm infection could not be demonstrated after adjustment for multiple comparisons. Further investigation of the equine gut microbiome and metabolome using larger studies is therefore warranted.

## Figures and Tables

**Table 1:** Taxa that were significantly different in abundance between tapeworm groups and controls and those with high and low FEC, irrespective of tapeworm infection.

Taxon	*p*-Value (FDR Corrected)	More Abundant in
**TP vs. CO**		
**Family**		
Bacteroidales UCG-001	0.016	CO
**Genus**		
Ruminococcaceae UCG-004	0.006	CO
*Jeotgalicoccus*	0.012	TP
*Candidatus Soleaferrea*	0.012	CO
*Romboutsia*	0.028	CO
**TP_21 vs. CO**	***p*** **-value** **(FDR corrected)**	**More abundant in**
**Order**		
Rickettsiales	0.004	CO
**Family**		
Rickettsiales Incertae Sedis	0.005	CO
**Genus**		
*Candidatus Hepatincola*	0.01	CO
*Selenomonas 3*	0.02	TP_21
**High_FEC vs. Low_FEC**	***p*** **-value** **(FDR corrected)**	**More abundant in**
**Order**		
Rhodospirillales	0.004	High_FEC
**Family**		
**Fusobacteriaceae**	0.003	High_FEC
**Rhodospirillaceae**	0.003	High_FEC
**Bacteroidaceae**	0.003	High_FEC
**Genus**		
***Bacteroides***	0.005	High_FEC
***Fusobacterium***	0.006	High_FEC
***Thalassospira***	0.007	High_FEC
***Gordonibacter***	0.021	High_FEC
***Prevotellaceae Ga6A1 group***	0.031	High_FEC

Differential abundance was calculated by the DESeq2 R package. Key: TP = tapeworm positive (n = 20), TP_21 = tapeworm samples with ≥21 worms (n = 9), CO = control (tapeworm negative, n = 28), Low_FEC = low strongyles (≤10 e.p.g), High_FEC = high strongyles (≥100 e.p.g). Key: FEC = faecal egg count, e.p.g = eggs per gram.

**Table 2:** Comparison of volatile organic compound (VOC) abundance from the colonic contents of horses positive for *Anoplocephala perfoliata* and non-infected controls.

TP vs. CO	*p*-Value	Adjusted *p*-Value	More Abundant in
**VOCs**			
Undecane, 2,6-dimethyl-	0.006	0.147	TP
Acetic acid	0.006	0.147	TP
Nonane	0.008	0.147	TP
Furan, 2-methyl-	0.009	0.147	TP
2-Undecanone	0.013	0.147	TP
1H-Pyrrole-2,5-dione, 3-ethyl-4-methyl-	0.013	0.147	TP
Dodecane, 2,6,10-trimethyl-	0.014	0.147	TP
3-Pentanone, 2-methyl-	0.015	0.147	TP
Heptane, 2-methyl-	0.017	0.147	TP
2-Heptanone, 6-methyl-	0.019	0.147	TP
2-Nonanone	0.020	0.147	TP
2-Octene, (E)-	0.025	0.147	TP
2-Decanone	0.026	0.147	TP
2-Octanone	0.026	0.147	TP
1-Penten-3-one	0.026	0.147	TP
Cyclohexanone, 2,2,6-trimethyl-	0.029	0.147	TP
3-Octanone	0.030	0.147	TP
D-Limonene	0.033	0.147	TP
2-Butanone	0.033	0.147	TP
Propanal	0.035	0.147	TP
Furan, 2-pentyl-	0.036	0.147	TP
2-Heptanone	0.038	0.147	TP
2-Butanone, 4-(2,6,6-trimethyl-1-cyclohexen-1-yl)-	0.042	0.156	TP
1-Cyclohexene-1-carboxaldehyde, 2,6,6-trimethyl-	0.044	0.156	TP
**TP_21 vs. CO**	*p*-value	Adjusted *p*-value	More abundant in
**VOCs**			
Undecane, 2,6-dimethyl-	0.001	0.127	TP_21
1H-Pyrrole-2,5-dione, 3-ethyl-4-methyl-	0.005	0.128	TP_21
Heptane, 2-methyl-	0.006	0.128	TP_21
2-Heptanone, 6-methyl-	0.006	0.128	TP_21
1-Cyclohexene-1-carboxaldehyde, 2,6,6-trimethyl-	0.012	0.174	TP_21
2-Undecanone	0.015	0.174	TP_21
Cyclohexanone, 2,2,6-trimethyl-	0.015	0.174	TP_21
2-Octene, (E)-	0.017	0.174	TP_21
Furan, 2-pentyl-	0.020	0.174	TP_21
Acetic acid	0.021	0.174	TP_21
Octane	0.023	0.178	TP_21
Nonane	0.027	0.189	TP_21
Decane	0.031	0.192	TP_21
Furan, 2-methyl-	0.034	0.192	TP_21
**TP_21 vs. CO**	*p*-value	Adjusted *p*-value	More abundant in
5-Hepten-2-one, 6-methyl-	0.036	0.192	TP_21
2-Decanone	0.036	0.192	TP_21
1-Octen-3-ol	0.041	0.203	TP_21
**High vs. Low FEC**			
**VOCs**			
1-Propanol, 2-methyl	0.0219	0.903	High_FEC
1-Hexanol	0.0381	0.903	High_FEC

Results are shown for VOCs which were significantly different in abundance (*p* < 0.05, *t*-test) prior to FDR and their corrected *p*-values. Key: TP = tapeworm positive (n = 20), TP_21 = tapeworm samples with ≥21 worms (n = 9), CO = control (tapeworm negative, n = 28), FEC = faecal egg count, High_FEC = ≥100 strongyle eggs per gram (n = 24), Low_FEC = ≤10 strongyle eggs per gram (n = 10).

**Table 3:** Operational taxonomic units (OTUs) and volatile organic compounds (VOCs) identified in single omics analysis that were significantly correlated with each other when integrated.

OTU	*p*-Value	OTU More Abundant in	VOCs Significantly Correlated	Correlation	VOC More Abundant in
OTU5074	<0.001	CO	Acetic acid	−0.42	TP
OTU3878	<0.001	TP	2-Nonanone	0.46	TP
			2-Decanone	0.43	TP
			1-Octen-3-ol	0.43	TP
OTU705	<0.001	CO	2-Octanone	−0.46	TP
			1-Octen-3-ol	−0.47	TP
OTU314	<0.001	TP	1H-Pyrrole-2,5-dione, 3-ethyl-4-methyl-	0.43	TP
			Cyclohexanone, 2,2,6-trimethyl-	0.41	TP
			Undecane, 2,6-dimethyl-	0.41	TP
			2-Heptanone, 6-methyl-	0.4	TP
			1-Cyclohexene-1-carboxaldehyde, 2,6,6-trimethyl-	0.4	TP
			Dodecane, 2,6,10-trimethyl-	0.37	TP
			Acetic acid	0.37	TP
OTU673	<0.001	CO	Heptane, 2-methyl-	−0.37	TP
OTU2331	<0.001	TP	2-Heptanone, 6-methyl-	0.6	TP
			Decane	0.58	TP
			Propanal	0.56	TP
			1-Cyclohexene-1-carboxaldehyde, 2,6,6-trimethyl-	0.55	TP
			Furan, 2-pentyl-	0.55	TP
			Cyclohexanone, 2,2,6-trimethyl-	0.53	TP
			Undecane, 2,6-dimethyl-	0.53	TP
			1-Octen-3-ol	0.51	TP
			1H-Pyrrole-2,5-dione, 3-ethyl-4-methyl-	0.48	TP
			2-Heptanone	0.45	TP
			Pentanal	0.45	TP
			2-Decanone	0.43	TP
			Furan, 2-methyl-	0.43	TP
			3-Octanone	0.42	TP
			2-Nonanone	0.4	TP
			2-Undecanone	0.4	TP
			Nonane	0.37	TP
OTU2051	<0.001	CO	Acetic acid	−0.37	TP
**OTU**	*p*-value	OTU more abundant in	VOCs significantly correlated	Correlation	VOC more abundant in
			1H-Pyrrole-2,5-dione, 3-ethyl-4-methyl-	−0.38	TP
			2-Heptanone	−0.39	TP
			1-Octen-3-ol	−0.41	TP
			2-Decanone	−0.45	TP
			2-Octanone	−0.47	TP
OTU142	0.015	CO	2-Nonanone	−0.37	TP
			Furan, 2-pentyl-	−0.38	TP
			2-Butanone, 4-(2,6,6-trimethyl-1-cyclohexen-1-yl)-	−0.4	TP
			2-Decanone	−0.43	TP
			Propanal	−0.43	TP
			2-Heptanone	−0.43	TP
			1-Octen-3-ol	−0.44	TP
OTU147	0.015	TP	2-Heptanone	0.56	TP
			3-Octanone	0.53	TP
			1H-Pyrrole-2,5-dione, 3-ethyl-4-methyl-	0.53	TP
			Furan, 2-pentyl-	0.53	TP
			2-Decanone	0.51	TP
			2-Nonanone	0.51	TP
			1-Penten-3-one	0.49	TP
			Nonane	0.48	TP
			2-Heptanone, 6-methyl-	0.47	TP
			2-Undecanone	0.47	TP
			1-Octen-3-ol	0.45	TP
			Cyclohexanone, 2,2,6-trimethyl-	0.43	TP
			2-Octanone	0.43	TP
			1-Cyclohexene-1-carboxaldehyde, 2,6,6-trimethyl-	0.42	TP
			Propanal	0.42	TP
			Furan, 2-methyl-	0.41	TP
			Dodecane, 2,6,10-trimethyl-	0.4	TP
			Acetic acid	0.39	TP
			D-limonene	0.36	TP
OTU239	0.020	CO	Decane	0.41	TP
			2-Heptanone, 6-methyl-	0.39	TP

OTUs were identified as significantly different between CO and TP by DeSeq2 R package. VOC abundances were identified as significantly different (pre-FDR) between CO and TP by *t*-test. Correlations between OTUs and VOCs were recorded from 0.3 to 1 (−0.3 to −1) to indicate positive (negative) linear relationships. The correlations were calculated from a Pearson’s correlation plot and the cor.test() function was used to determine if correlations were statistically significant. OTU classifications: OTU5074 Treponema 2, OTU3878 Alloprevotella, OTU705 Bacteroidales S24-7 group, OTU314 (Eubacterium) oxidoreducens group, OTU673 Lachnospiraceae NK4A136 group, OTU2331 Prevotellaceae UCG-003, OTU2051 Rikenellaceae RC9 gut group, OTU142 Ruminococcaceae UCG-005, OTU147 Prevotellaceae UCG-001, OTU239 Prevotella 1. CO = control (tapeworm negative, n = 28), TP = tapeworm positive (n = 20).

## Data Availability

The raw microbiome and metabolome data are available at https://data.mendeley.com/datasets/44sn7kbn6x/1 (accessed on 29 November 2020).

## Supplementary Materials

The following are available online at https://www.mdpi.com/2076-2615/11/3/755/s1, Table S1: Parasitology results of horses included in the study, Table S2: Barcoded index primers, Table S3: A list of VOCs identified and frequencies of occurrence in TP and CO samples, Table S4: Taxonomic identification of OTUs in Figure 6., Figure S1: Comparison of VOC numbers and chromatogram of colon contents freeze-dried (FD) and not freeze-dried (NFD), Figure S2: (**A**) Significant OTUs comparing TP and CO. (**B**) Significant OTUs comparing TP_21 and CO., Figure S3 Significant OTUs between Low_FEC and High_FEC groups, Figure S4: (**A**) PCA of VOC profile (tapeworm positive = TP and tapeworm negative = CO), (**B**) PCA of VOC profile (≥21 tapeworms = TP_21 and tapeworm negative = CO), Figure S5: Balanced error rate for mixOmics model.
