# Supplementary material for: A Comparison of the Colonic Microbiome and Volatile Organic Compound Metabolome of Anoplocephala perfoliata Infected and Non-Infected Horses: A Pilot Study
Source: Animals (Basel). 2021 Mar 9;11(3):755. doi: 10.3390/ani11030755 (PMC7999024; doi:10.3390/ani11030755)
Supplement: Supplementary file 1 [file animals-11-00755-s001.pdf]

Supplementary materials

Table S1. Parasitology results of horses included in the study. Key: FEC = faecal egg count, e.p.g = eggs per gram. Ascarid eggs were also checked for, but all samples were negative.

**Horse ID (Control)**	**FEC (e.p.g)**	**Horse ID (Tapeworm positive)**	***A. perfoliata* status**	**FEC (e.p.g)**
C1	10	T1	low	90
C2	423	T2	Positive, unrecorded	19
C3	141	T3	low	0
C4	42	T4	low	12
C5	156	T5	low	273
C6	180	T7	med	22
C7	0	T8	low	300
C8	72	T9	Positive, unrecorded	177
C9	234	T10	Positive, unrecorded	105
C10	135	T11	Positive, unrecorded	162
C12	0	T12	high	43
C13	23	T13	high	48
C14	174	T14	med	315
C15	135	T15	high	54
C16	288	T16	high	31
C17	159	T17	med	66
C18	219	T18	high	0
C19	252	T19	med	135
C20	174	T20	low	2
C21	0	T21	low	1
C22	120			
C23	0			
C24	204			
C26	0			
C27	13			
C28	216			
C29	99			
C30	282			

Table S2. Barcoded index primers.

Sample	Primer set	Forward	Reverse
Overhang region on first primer set		ACA CTC TTT CCC TAC ACG ACG CTC TTC CGA TCT	GTG ACT GGA GTT CAG ACG TGT GCT CTT CCG ATC T
Neg	N502_N701	AATGATACGGCGACCACCGAGATCTACACCTCTCTATACACTCTTTCCCTACACGACGCTC	CAAGCAGAAGACGGCATACGAGATTCGCCTTAGTGACTGGAGTTCAGACGTGTGCTC
T5	N502_N702	AATGATACGGCGACCACCGAGATCTACACCTCTCTATACACTCTTTCCCTACACGACGCTC	CAAGCAGAAGACGGCATACGAGATCTAGTACGGTGACTGGAGTTCAGACGTGTGCTC
C19	N502_N703	AATGATACGGCGACCACCGAGATCTACACCTCTCTATACACTCTTTCCCTACACGACGCTC	CAAGCAGAAGACGGCATACGAGATTTCTGCCTGTGACTGGAGTTCAGACGTGTGCTC
C14	N502_N704	AATGATACGGCGACCACCGAGATCTACACCTCTCTATACACTCTTTCCCTACACGACGCTC	CAAGCAGAAGACGGCATACGAGATGCTCAGGAGTGACTGGAGTTCAGACGTGTGCTC
C16	N502_N705	AATGATACGGCGACCACCGAGATCTACACCTCTCTATACACTCTTTCCCTACACGACGCTC	CAAGCAGAAGACGGCATACGAGATAGGAGTCCGTGACTGGAGTTCAGACGTGTGCTC
T7	N502_N706	AATGATACGGCGACCACCGAGATCTACACCTCTCTATACACTCTTTCCCTACACGACGCTC	CAAGCAGAAGACGGCATACGAGATCATGCCTAGTGACTGGAGTTCAGACGTGTGCTC
T10	N502_N707	AATGATACGGCGACCACCGAGATCTACACCTCTCTATACACTCTTTCCCTACACGACGCTC	CAAGCAGAAGACGGCATACGAGATGTAGAGAGGTGACTGGAGTTCAGACGTGTGCTC
C20	N502_N708	AATGATACGGCGACCACCGAGATCTACACCTCTCTATACACTCTTTCCCTACACGACGCTC	CAAGCAGAAGACGGCATACGAGATCCTCTCTGGTGACTGGAGTTCAGACGTGTGCTC
C2	N502_N709	AATGATACGGCGACCACCGAGATCTACACCTCTCTATACACTCTTTCCCTACACGACGCTC	CAAGCAGAAGACGGCATACGAGATAGCGTAGCGTGACTGGAGTTCAGACGTGTGCTC
C6	N503_N702	AATGATACGGCGACCACCGAGATCTACACTATCCTCTACACTCTTTCCCTACACGACGCTC	CAAGCAGAAGACGGCATACGAGATCTAGTACGGTGACTGGAGTTCAGACGTGTGCTC
C30	N503_N703	AATGATACGGCGACCACCGAGATCTACACTATCCTCTACACTCTTTCCCTACACGACGCTC	CAAGCAGAAGACGGCATACGAGATTTCTGCCTGTGACTGGAGTTCAGACGTGTGCTC
C15	N503_N705	AATGATACGGCGACCACCGAGATCTACACTATCCTCTACACTCTTTCCCTACACGACGCTC	CAAGCAGAAGACGGCATACGAGATAGGAGTCCGTGACTGGAGTTCAGACGTGTGCTC
T21	N503_N706	AATGATACGGCGACCACCGAGATCTACACTATCCTCTACACTCTTTCCCTACACGACGCTC	CAAGCAGAAGACGGCATACGAGATCATGCCTAGTGACTGGAGTTCAGACGTGTGCTC
T11	N503_N707	AATGATACGGCGACCACCGAGATCTACACTATCCTCTACACTCTTTCCCTACACGACGCTC	CAAGCAGAAGACGGCATACGAGATGTAGAGAGGTGACTGGAGTTCAGACGTGTGCTC
T8	N503_N708	AATGATACGGCGACCACCGAGATCTACACTATCCTCTACACTCTTTCCCTACACGACGCTC	CAAGCAGAAGACGGCATACGAGATCCTCTCTGGTGACTGGAGTTCAGACGTGTGCTC
T1	N503_N709	AATGATACGGCGACCACCGAGATCTACACTATCCTCTACACTCTTTCCCTACACGACGCTC	CAAGCAGAAGACGGCATACGAGATAGCGTAGCGTGACTGGAGTTCAGACGTGTGCTC
T20	N503_N710	AATGATACGGCGACCACCGAGATCTACACTATCCTCTACACTCTTTCCCTACACGACGCTC	CAAGCAGAAGACGGCATACGAGATCAGCCTCGGTGACTGGAGTTCAGACGTGTGCTC
C22	N504_N702	AATGATACGGCGACCACCGAGATCTACACAGAGTAGAACACTCTTTCCCTACACGACGCTC	CAAGCAGAAGACGGCATACGAGATCTAGTACGGTGACTGGAGTTCAGACGTGTGCTC
C26	N504_N703	AATGATACGGCGACCACCGAGATCTACACAGAGTAGAACACTCTTTCCCTACACGACGCTC	CAAGCAGAAGACGGCATACGAGATTTCTGCCTGTGACTGGAGTTCAGACGTGTGCTC
T13	N504_N704	AATGATACGGCGACCACCGAGATCTACACAGAGTAGAACACTCTTTCCCTACACGACGCTC	CAAGCAGAAGACGGCATACGAGATGCTCAGGAGTGACTGGAGTTCAGACGTGTGCTC

Sample	Primer set	Forward	Reverse
T4	N504_N705	AATGATACGGCGACCACCGAGATCTACACAGAGTAGAACACTCTTTCCCTACACGACGCTC	CAAGCAGAAGACGGCATACGAGATAGGAGTCCGTGACTGGAGTTCAGACGTGTGCTC
C9	N504_N706	AATGATACGGCGACCACCGAGATCTACACAGAGTAGAACACTCTTTCCCTACACGACGCTC	CAAGCAGAAGACGGCATACGAGATCATGCCTAGTGACTGGAGTTCAGACGTGTGCTC
C18	N504_N707	AATGATACGGCGACCACCGAGATCTACACAGAGTAGAACACTCTTTCCCTACACGACGCTC	CAAGCAGAAGACGGCATACGAGATGTAGAGAGGTGACTGGAGTTCAGACGTGTGCTC
T3	N504_N708	AATGATACGGCGACCACCGAGATCTACACAGAGTAGAACACTCTTTCCCTACACGACGCTC	CAAGCAGAAGACGGCATACGAGATCCTCTCTGGTGACTGGAGTTCAGACGTGTGCTC
C17	N504_N709	AATGATACGGCGACCACCGAGATCTACACAGAGTAGAACACTCTTTCCCTACACGACGCTC	CAAGCAGAAGACGGCATACGAGATAGCGTAGCGTGACTGGAGTTCAGACGTGTGCTC
C1	N504_N710	AATGATACGGCGACCACCGAGATCTACACAGAGTAGAACACTCTTTCCCTACACGACGCTC	CAAGCAGAAGACGGCATACGAGATCAGCCTCGGTGACTGGAGTTCAGACGTGTGCTC
C13	N505_N702	AATGATACGGCGACCACCGAGATCTACACGTAAGGAGACACTCTTTCCCTACACGACGCTC	CAAGCAGAAGACGGCATACGAGATCTAGTACGGTGACTGGAGTTCAGACGTGTGCTC
T12	N505_N703	AATGATACGGCGACCACCGAGATCTACACGTAAGGAGACACTCTTTCCCTACACGACGCTC	CAAGCAGAAGACGGCATACGAGATTTCTGCCTGTGACTGGAGTTCAGACGTGTGCTC
C10	N505_N704	AATGATACGGCGACCACCGAGATCTACACGTAAGGAGACACTCTTTCCCTACACGACGCTC	CAAGCAGAAGACGGCATACGAGATGCTCAGGAGTGACTGGAGTTCAGACGTGTGCTC
T15	N505_N705	AATGATACGGCGACCACCGAGATCTACACGTAAGGAGACACTCTTTCCCTACACGACGCTC	CAAGCAGAAGACGGCATACGAGATAGGAGTCCGTGACTGGAGTTCAGACGTGTGCTC
T19	N505_N706	AATGATACGGCGACCACCGAGATCTACACGTAAGGAGACACTCTTTCCCTACACGACGCTC	CAAGCAGAAGACGGCATACGAGATCATGCCTAGTGACTGGAGTTCAGACGTGTGCTC
T14	N505_N707	AATGATACGGCGACCACCGAGATCTACACGTAAGGAGACACTCTTTCCCTACACGACGCTC	CAAGCAGAAGACGGCATACGAGATGTAGAGAGGTGACTGGAGTTCAGACGTGTGCTC
C23	N505_N708	AATGATACGGCGACCACCGAGATCTACACGTAAGGAGACACTCTTTCCCTACACGACGCTC	CAAGCAGAAGACGGCATACGAGATCCTCTCTGGTGACTGGAGTTCAGACGTGTGCTC
C3	N505_N709	AATGATACGGCGACCACCGAGATCTACACGTAAGGAGACACTCTTTCCCTACACGACGCTC	CAAGCAGAAGACGGCATACGAGATAGCGTAGCGTGACTGGAGTTCAGACGTGTGCTC
T9	N506_N702	AATGATACGGCGACCACCGAGATCTACACACTGCATAACACTCTTTCCCTACACGACGCTC	CAAGCAGAAGACGGCATACGAGATCTAGTACGGTGACTGGAGTTCAGACGTGTGCTC
T17	N506_N703	AATGATACGGCGACCACCGAGATCTACACACTGCATAACACTCTTTCCCTACACGACGCTC	CAAGCAGAAGACGGCATACGAGATTTCTGCCTGTGACTGGAGTTCAGACGTGTGCTC
C28	N506_N704	AATGATACGGCGACCACCGAGATCTACACACTGCATAACACTCTTTCCCTACACGACGCTC	CAAGCAGAAGACGGCATACGAGATGCTCAGGAGTGACTGGAGTTCAGACGTGTGCTC
C21	N506_N705	AATGATACGGCGACCACCGAGATCTACACACTGCATAACACTCTTTCCCTACACGACGCTC	CAAGCAGAAGACGGCATACGAGATAGGAGTCCGTGACTGGAGTTCAGACGTGTGCTC
C4	N506_N706	AATGATACGGCGACCACCGAGATCTACACACTGCATAACACTCTTTCCCTACACGACGCTC	CAAGCAGAAGACGGCATACGAGATCATGCCTAGTGACTGGAGTTCAGACGTGTGCTC
T2	N506_N707	AATGATACGGCGACCACCGAGATCTACACACTGCATAACACTCTTTCCCTACACGACGCTC	CAAGCAGAAGACGGCATACGAGATGTAGAGAGGTGACTGGAGTTCAGACGTGTGCTC
C27	N506_N708	AATGATACGGCGACCACCGAGATCTACACACTGCATAACACTCTTTCCCTACACGACGCTC	CAAGCAGAAGACGGCATACGAGATCCTCTCTGGTGACTGGAGTTCAGACGTGTGCTC
C12	N506_N709	AATGATACGGCGACCACCGAGATCTACACACTGCATAACACTCTTTCCCTACACGACGCTC	CAAGCAGAAGACGGCATACGAGATAGCGTAGCGTGACTGGAGTTCAGACGTGTGCTC

C5	N507_N702	AATGATACGGCGACCACCGAGATCTACACAAGGAGTAACACTCTTTCCCTACACGACGCTC	CAAGCAGAAGACGGCATACGAGATCTAGTACGGTGACTGGAGTTCAGACGTGTGCTC
C24	N507_N703	AATGATACGGCGACCACCGAGATCTACACAAGGAGTAACACTCTTTCCCTACACGACGCTC	CAAGCAGAAGACGGCATACGAGATTTCTGCCTGTGACTGGAGTTCAGACGTGTGCTC
T16	N507_N704	AATGATACGGCGACCACCGAGATCTACACAAGGAGTAACACTCTTTCCCTACACGACGCTC	CAAGCAGAAGACGGCATACGAGATGCTCAGGAGTGACTGGAGTTCAGACGTGTGCTC
T18	N507_N706	AATGATACGGCGACCACCGAGATCTACACAAGGAGTAACACTCTTTCCCTACACGACGCTC	CAAGCAGAAGACGGCATACGAGATCATGCCTAGTGACTGGAGTTCAGACGTGTGCTC
C7	N507_N707	AATGATACGGCGACCACCGAGATCTACACAAGGAGTAACACTCTTTCCCTACACGACGCTC	CAAGCAGAAGACGGCATACGAGATGTAGAGAGGTGACTGGAGTTCAGACGTGTGCTC
C29	N507_N708	AATGATACGGCGACCACCGAGATCTACACAAGGAGTAACACTCTTTCCCTACACGACGCTC	CAAGCAGAAGACGGCATACGAGATCCTCTCTGGTGACTGGAGTTCAGACGTGTGCTC
C8	N507_N709	AATGATACGGCGACCACCGAGATCTACACAAGGAGTAACACTCTTTCCCTACACGACGCTC	CAAGCAGAAGACGGCATACGAGATAGCGTAGCGTGACTGGAGTTCAGACGTGTGCTC

Table S3. A list of VOCs identified and frequencies of occurrence in TP and CO samples.

Retention time_VOC	Present Control	Present Tape
10.22_2,3-Butanedione	28	20
10.50_2-Butanone	21	20
10.60_Ethyl acetate	23	13
10.80_2-Butanol	24	17
11.93_1-Propanol, 2-methyl-	27	17
12.45_Butanal, 3-methyl-	28	20
12.55_Acetic acid	11	14
12.79_Butanal, 2-methyl-	28	20
13.43_Furan, 2-ethyl-	20	16
13.80_1-Penten-3-one	5	10
13.88_2-Pentanone	21	16
13.94_1-Penten-3-ol	28	18
14.21_Pentanal	28	20
14.30_n-Propyl acetate	16	15
14.63_Butanoic acid, methyl ester	25	17
15.21_Heptane, 2-methyl-	12	15
16.07_Propanoic acid	19	17
16.19_3-Pentanone, 2-methyl-	11	13
16.50_Toluene	25	19
16.66_Octane	27	19
16.92_2-Octene, (E)-	4	10
16.93_1-Propanone, 1-cyclopropyl-	26	17
17.27_1-Pentanol	26	19
17.61_Butanoic acid, ethyl ester	24	16
18.01_2-Hexanone	23	17
18.25_Hexanal	28	20
18.64_Methyl valerate	13	11
19.40_Butanoic acid	25	19
20.30_Ethylbenzene	27	20
20.49_Nonane	28	20
20.60_Benzene, 1,3-dimethyl-	28	20
21.00_2-Hexenal	23	17
21.32_1-Hexanol	14	11
21.71_Styrene	28	19
21.73_Butanoic acid, 2-methyl-	6	9
21.94_2-Heptanone	28	20
22.05_1,7-Octadiene, 2,7-dimethyl-	25	16
22.26_Heptanal	28	20
22.52_1,6-Octadiene, 3,7-dimethyl-, (S)-	27	20
22.85_Nonane, 2-methyl-	22	17
24.21_Decane	28	20
24.37_2-Heptanone, 6-methyl-	28	20
24.68_2-Heptanone, 5-methyl-	21	16
24.74_Furan, 2-pentyl-	28	19
24.90_1-Heptanol	5	11
25.23_1-Octen-3-ol	25	19
25.36_3-Octanone	27	20
25.44_Benzene, 1,2,4-trimethyl-	18	13
25.441_Benzaldehyde	15	10
25.55_5-Hepten-2-one, 6-methyl-	28	20
25.66_2-Octanone	12	14
25.68_3-Octanol	26	17
26.01_Octanal	28	20
26.20_D-Limonene	28	20
26.53_Dimethyl sulfone	28	20
27.66_Cyclohexanone, 2,2,6-trimethyl-	27	20
27.70_Undecane	15	9

28.00_Phenol	25	19
29.16_2-Nonanone	16	15
29.51_Nonanal	25	17
30.37_2(3H)-Furanone, 5-ethyldihydro-	20	14
30.75_Dodecane	27	20
30.91_p-Cresol	17	14
31.43_Undecane, 2,6-dimethyl-	27	20
31.50_Benzyl methyl ketone	9	9
32.41_2-Decanone	27	20
34.03_Tridecane	27	20
34.15_1-Cyclohexene-1-carboxaldehyde, 2,6,6-trimethyl-	28	20
35.46_2-Undecanone	22	18
35.73_1H-Pyrrole-2,5-dione, 3-ethyl-4-methyl-	9	11
36.01_Tridecane, 3-methyl-	21	16
36.30_Dodecane, 2,6,10-trimethyl-	28	20
36.89_Tetradecane	28	20
38.70_Heptadecane, 2,6,10,14-tetramethyl-	28	20
40.72_2-Butanone, 4-(2,6,6-trimethyl-1-cyclohexen-1-yl)-	8	10
6.62_Ethanol	28	20
7.19_Propanal	28	20
7.35_Acetone	28	20
7.48_Dimethyl sulfide	21	12
7.55_Isopropyl Alcohol	19	11
7.92_Acetic acid, methyl ester	28	20
8.98_Propanal, 2-methyl-	28	20
9.35_Methacrolein	6	10
9.54_1-Propanol	27	20
9.89_Furan, 2-methyl-	12	14

Table S4. Taxonomic identification of OTUs in Fig 6.

OTU	Kingdom	Phylum	Class	Order	Family
**OTU1359**	Bacteria	Firmicutes	Clostridia	Clostridiales	Ruminococcaceae
**OTU2243**	Bacteria	Firmicutes	Clostridia	Clostridiales	Christensenellaceae
**OTU1127**	Bacteria	Firmicutes	Clostridia	Clostridiales	Lachnospiraceae
**OTU2331**	Bacteria	Bacteroidetes	Bacteroidia	Bacteroidales	Prevotellaceae
**OTU43**	Bacteria	Firmicutes	Clostridia	Clostridiales	Christensenellaceae
**OTU743**	Bacteria	Firmicutes	Clostridia	Clostridiales	Lachnospiraceae
**OTU3249**	Bacteria	Firmicutes	Clostridia	Clostridiales	Lachnospiraceae
**OTU298**	Bacteria	Firmicutes	Clostridia	Clostridiales	Ruminococcaceae
**OTU147**	Bacteria	Bacteroidetes	Bacteroidia	Bacteroidales	Prevotellaceae
**OTU2051**	Bacteria	Bacteroidetes	Bacteroidia	Bacteroidales	Rikenellaceae
**OTU980**	Bacteria	Firmicutes	Clostridia	Clostridiales	Lachnospiraceae
**OTU768**	Bacteria	Spirochaetae	Spirochaetes	Spirochaetales	Spirochaetaceae
**OTU2081**	Bacteria	Firmicutes	Clostridia	Clostridiales	Lachnospiraceae
**OTU776**	Bacteria	Firmicutes	Clostridia	Clostridiales	Christensenellaceae
**OTU720**	Bacteria	Firmicutes	Erysipelotrichia	Erysipelotrichales	Erysipelotrichaceae
**OTU1446**	Bacteria	Firmicutes	Clostridia	Clostridiales	Lachnospiraceae
**OTU526**	Bacteria	Bacteroidetes	Bacteroidia	Bacteroidales	Prevotellaceae

a)

b)

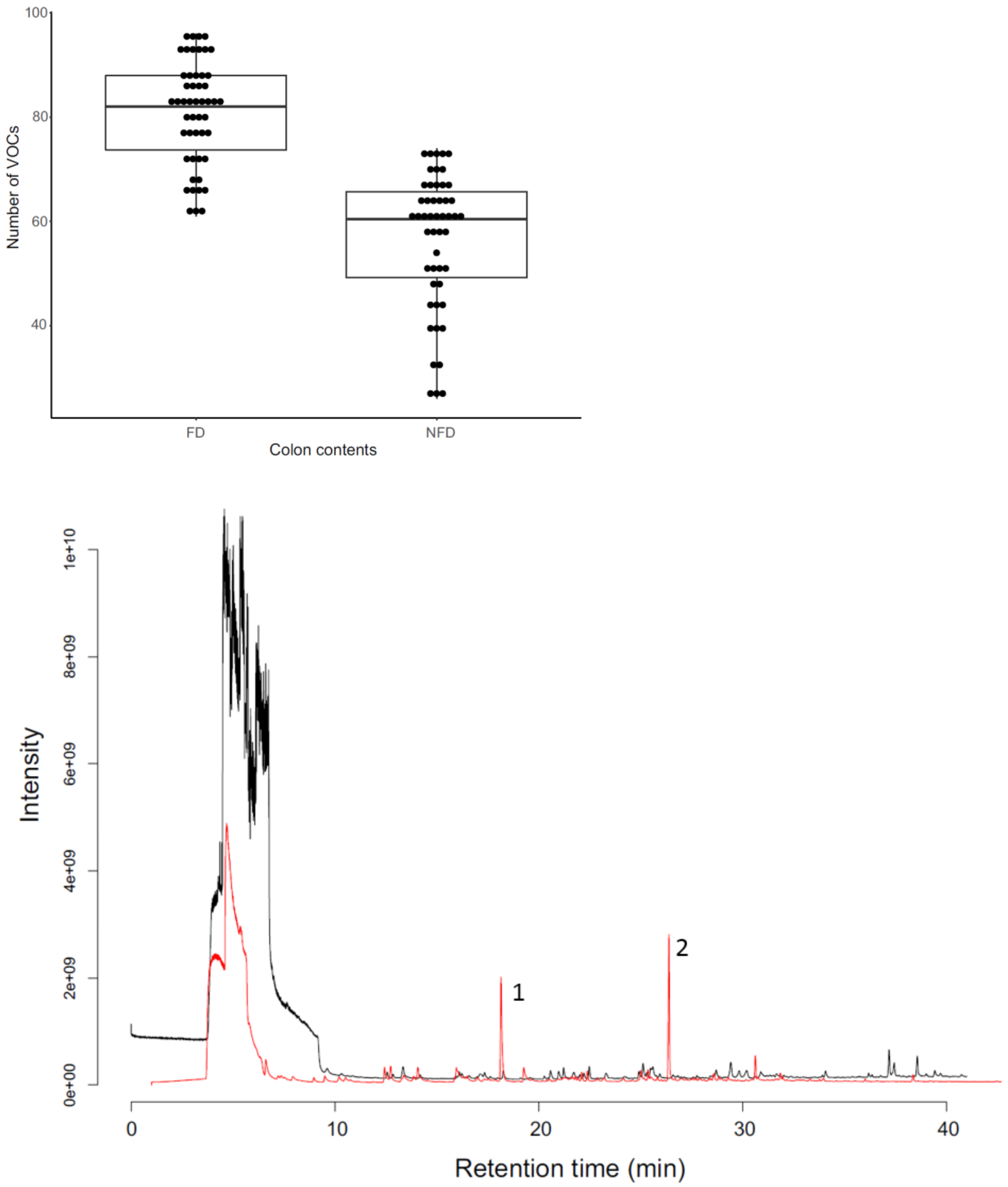

Figure S1. Comparison of VOC numbers and chromatogram of colon contents freeze-dried (FD) (n=50) and not freeze-dried (NFD) (n=50). One horse (C25) was excluded from the freeze-dry analysis as the sample failed to freeze-dry adequately. a) shows a boxplot of VOC numbers found in FD and NFD, a significantly greater number of VOCs were detected in FD rather than NFD (Wilcoxon signed-rank test with continuity correction, $p<0.001$). In b) a GCMS chromatogram overlay of the VOC profile of NFD (black trace) and FD (red trace) colon contents from one horse. Peak 1 = hexanal, peak 2 = dimethyl sulfone.

(A)

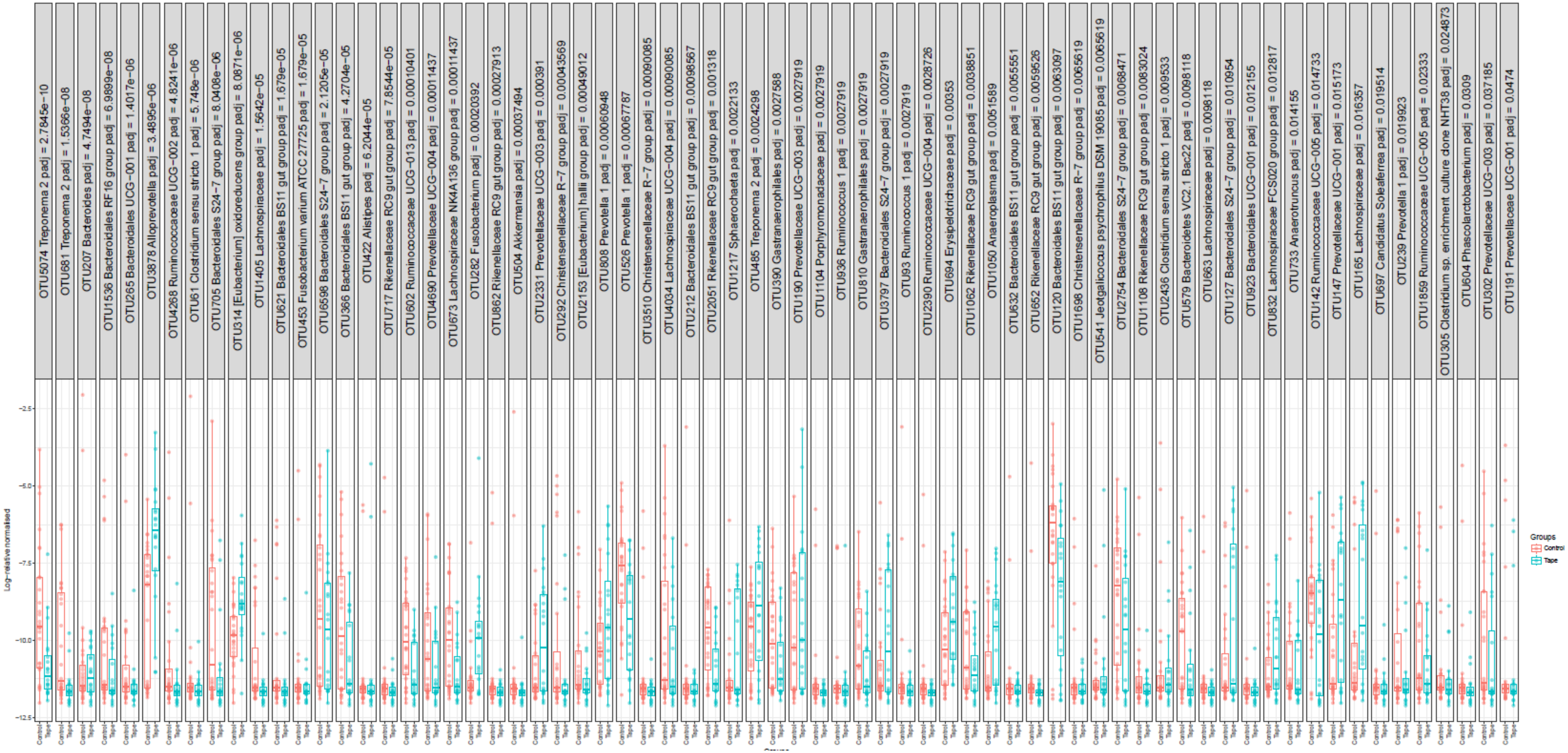

OTU5074 Treponema 2 padj = 2.7845e-10
OTU681 Treponema 2 padj = 1.5366e-08
OTU207 Bacteroides padj = 4.7494e-08
OTU1536 Bacteroidales RF16 group padj = 6.9899e-08
OTU265 Bacteroidales UCG-001 padj = 1.4017e-06
OTU3878 Alloprevotella padj = 3.4895e-06
OTU4268 Ruminococcaceae UCG-002 padj = 4.8241e-06
OTU61 Clostridium sensu stricto 1 padj = 5.748e-06
OTU705 Bacteroidales S24-7 group padj = 8.0408e-06
OTU314 [Eubacterium] oxidoreducens group padj = 8.0871e-06
OTU1405 Lachnospiraceae padj = 1.5642e-05
OTU621 Bacteroidales BS11 gut group padj = 1.679e-05
OTU453 Fusobacterium varium ATCC 27725 padj = 1.679e-05
OTU6598 Bacteroidales S24-7 group padj = 2.1205e-05
OTU366 Bacteroidales BS11 gut group padj = 4.2704e-05
OTU422 Alistipes padj = 6.2044e-05
OTU717 Rikenellaceae RC9 gut group padj = 7.8544e-05
OTU602 Ruminococcaceae UCG-013 padj = 0.00010401
OTU4690 Prevotellaceae UCG-004 padj = 0.00011437
OTU673 Lachnospiraceae NK4A136 group padj = 0.00011437
OTU282 Fusobacterium padj = 0.00020392
OTU862 Rikenellaceae RC9 gut group padj = 0.00027913
OTU504 Akkermansia padj = 0.00037494
OTU2331 Prevotellaceae UCG-003 padj = 0.000391
OTU292 Christensenellaceae R-7 group padj = 0.00043569
OTU2153 [Eubacterium] hallii group padj = 0.00049012
OTU808 Prevotella 1 padj = 0.00060948
OTU526 Prevotella 1 padj = 0.00067787
OTU3510 Christensenellaceae R-7 group padj = 0.00090085
OTU4034 Lachnospiraceae UCG-004 padj = 0.00090085
OTU212 Bacteroidales BS11 gut group padj = 0.00098567
OTU2051 Rikenellaceae RC9 gut group padj = 0.001318
OTU1217 Sphaerochaeta padj = 0.0022133
OTU485 Treponema 2 padj = 0.0024298
OTU390 Gastranaerophilales padj = 0.0027588
OTU190 Prevotellaceae UCG-003 padj = 0.0027919
OTU1104 Porphyromonadaceae padj = 0.0027919
OTU936 Ruminococcus 1 padj = 0.0027919
OTU810 Gastranaerophilales padj = 0.0027919
OTU3797 Bacteroidales S24-7 group padj = 0.0027919
OTU93 Ruminococcus 1 padj = 0.0027919
OTU2390 Ruminococcaceae UCG-004 padj = 0.0028726
OTU694 Erysipelotrichaceae padj = 0.00353
OTU1062 Rikenellaceae RC9 gut group padj = 0.0038851
OTU1050 Anaeroplasma padj = 0.0051589
OTU632 Bacteroidales BS11 gut group padj = 0.0055551
OTU652 Rikenellaceae RC9 gut group padj = 0.0059526
OTU120 Bacteroidales BS11 gut group padj = 0.0063097
OTU1698 Christensenellaceae R-7 group padj = 0.0065619
OTU541 Jeotgalicoccus psychrophilus DSM 19085 padj = 0.0065619
OTU2754 Bacteroidales S24-7 group padj = 0.0068471
OTU1108 Rikenellaceae RC9 gut group padj = 0.0083024
OTU2436 Clostridium sensu stricto 1 padj = 0.009533
OTU579 Bacteroidetes VC2.1 Bac22 padj = 0.0098118
OTU663 Lachnospiraceae padj = 0.0098118
OTU127 Bacteroidales S24-7 group padj = 0.010954
OTU923 Bacteroidales UCG-001 padj = 0.012155
OTU832 Lachnospiraceae FCS020 group padj = 0.012817
OTU733 Anaerotruncus padj = 0.014155
OTU142 Ruminococcaceae UCG-005 padj = 0.014733
OTU147 Prevotellaceae UCG-001 padj = 0.015173
OTU165 Lachnospiraceae padj = 0.016357
OTU697 Candidatus Soleaferrea padj = 0.019514
OTU239 Prevotella 1 padj = 0.019923
OTU1859 Ruminococcaceae UCG-005 padj = 0.02333
OTU305 Clostridium sp. enrichment culture clone NHT38 padj = 0.024873
OTU604 Phascolarctobacterium padj = 0.0309
OTU302 Prevotellaceae UCG-003 padj = 0.037185
OTU191 Prevotellaceae UCG-001 padj = 0.0474
Log-relative normalised
-2.5
-5.0
-7.5
-10.0
-12.5
Groups
Control
Tape

(B)

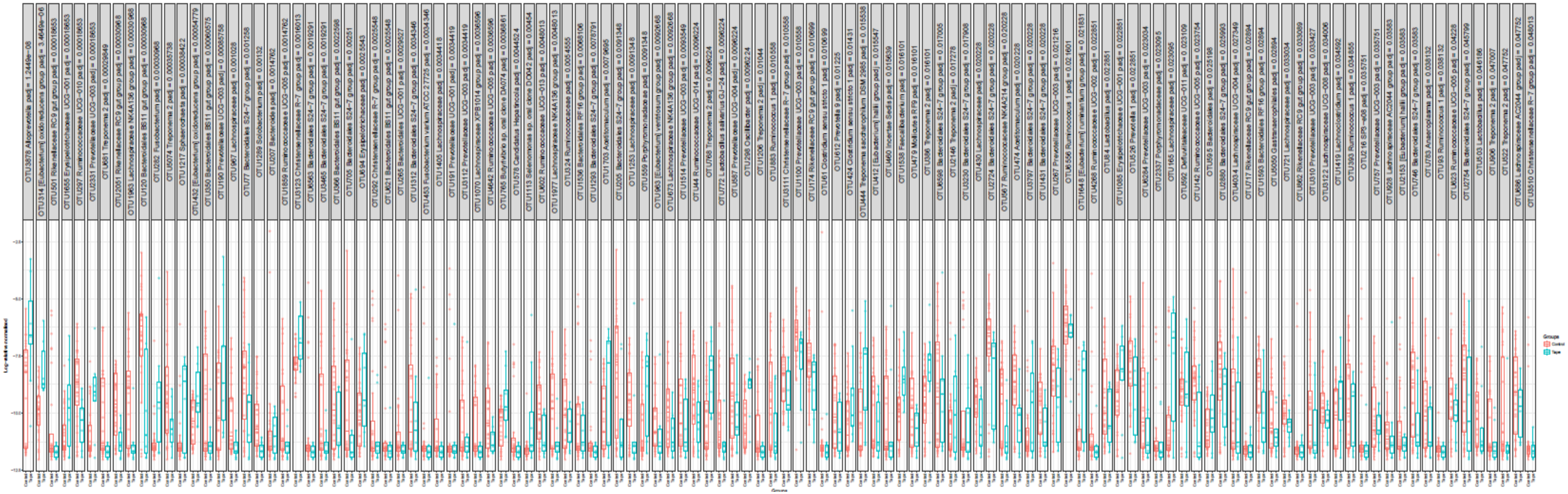

Figure S2. (A) Significant OTUs comparing TP and CO. **(B)** Significant OTUs comparing TP_21 and CO.

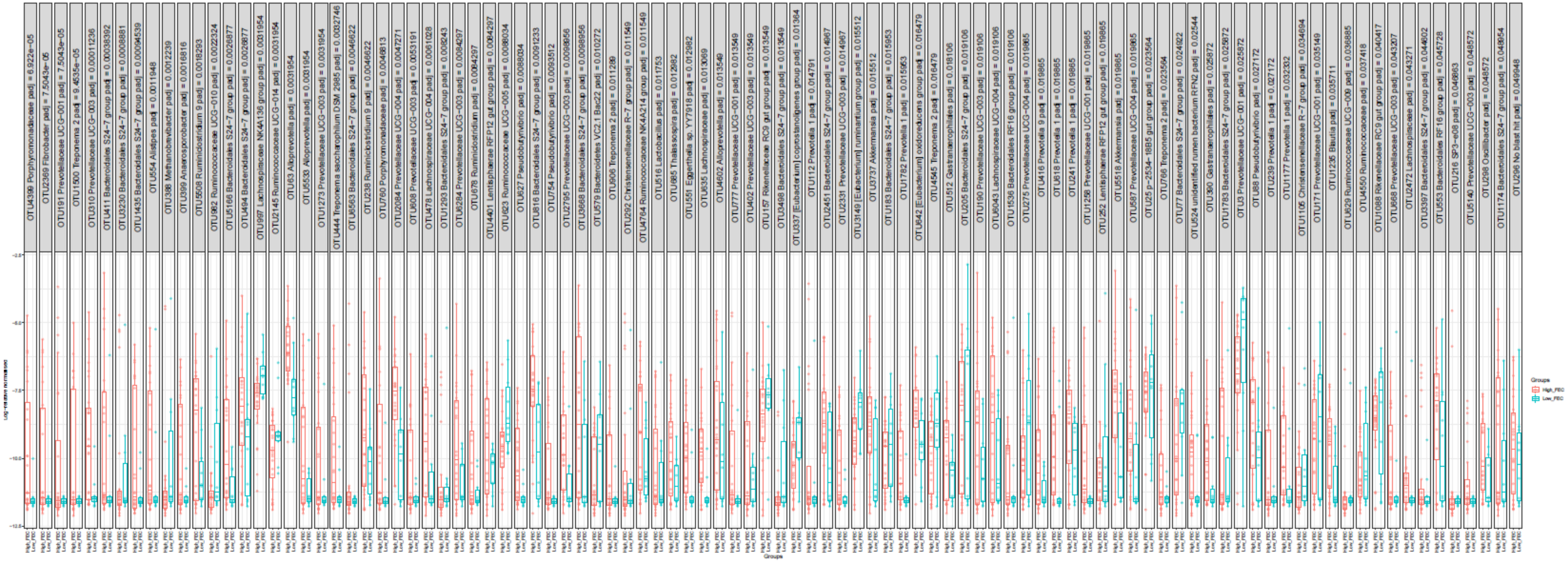

Figure S3. Significant OTUs between Low_FEC and High_FEC groups.

(A)

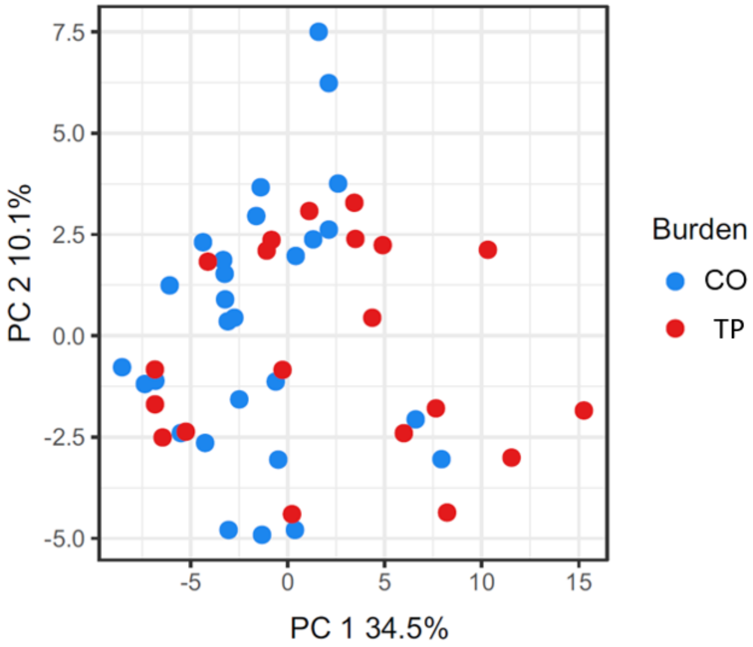

(B)

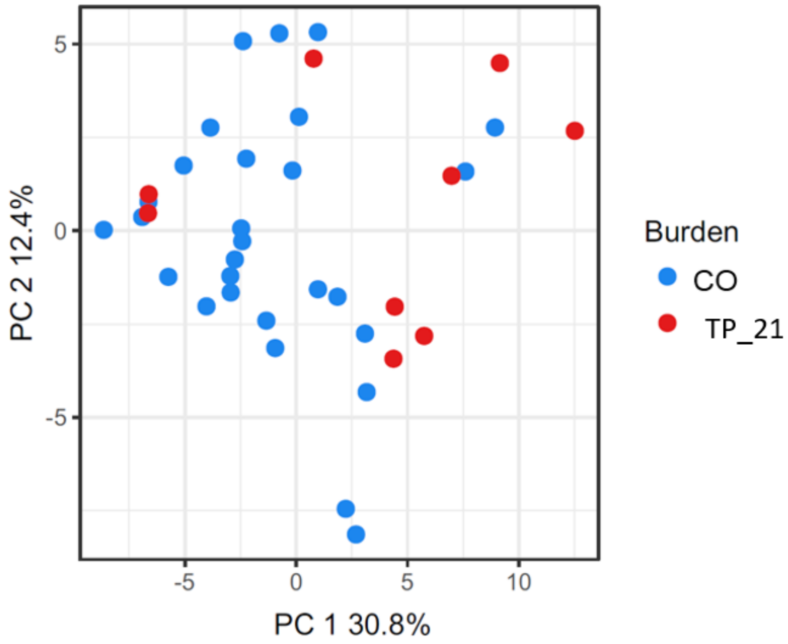

Figure S4. (**A**) PCA of VOC profile (tapeworm positive = TP and tapeworm negative = CO). (**B**) PCA of VOC profile (≥ 21 tapeworms = TP_21 and tapeworm negative = CO).

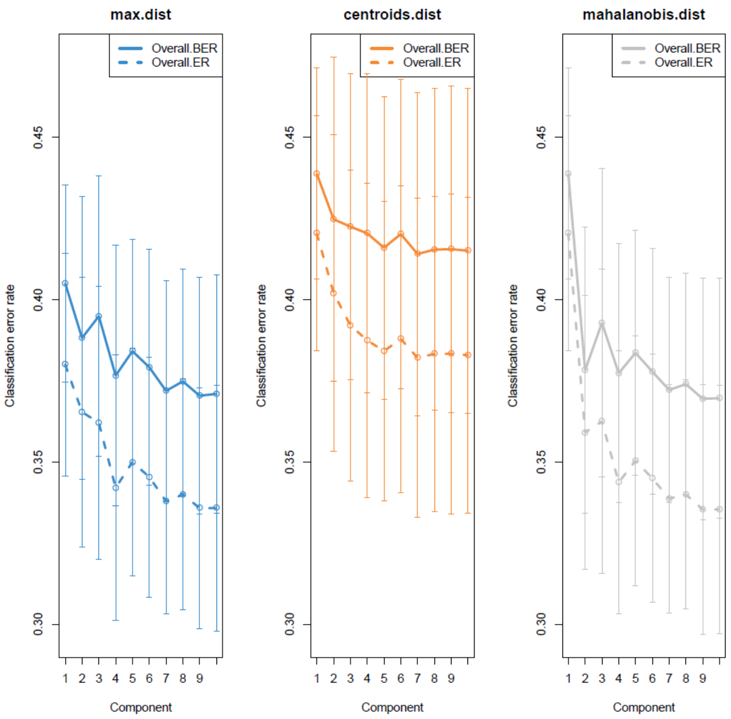

Figure S5. Balanced error rate for mixOmics model.
